# Diagnostic Pitfalls of Dental Follicles and Cyst-like Lesions in Juvenile Patients: An Early Odontogenic Myxoma Mimicking a Follicular Cyst

**DOI:** 10.3390/jcm15020599

**Published:** 2026-01-12

**Authors:** Kamil Nelke, Klaudiusz Łuczak, Michał Gontarz, Grażyna Wyszyńska-Pawelec, Agata Małyszek, Ömer Uranbey, Dayel Gerardo Rosales Díaz Mirón, Maciej Dobrzyński, Małgorzata Tarnowska, Piotr Kuropka

**Affiliations:** 1 Maxillo-Facial Surgery Ward, EMC Hospital, Pilczycka 144 Street, 54-144 Wrocław, Poland; 2 Academy of Applied Sciences, Health Department, Academy of Silesius in Wałbrzych, Zamkowa 4 Street, 58-300 Wałbrzych, Poland; 3 Department of Cranio-Maxillo-Facial Surgery, Maxillo-Facial Surgery Clinic, University Hospital in Cracow, Macieja Jakubowskiego 2 Street, (Nowy Prokocim), 30-688 Kraków, Poland; 4 Department of Biostructure and Animal Physiology, Wrocław University of Environmental and Life Sciences, Cypriana K. Norwida 31 Street, 50-375 Wrocław, Poland; 5 Faculty of Dentistry, Department of Oral and Maxillofacial Surgery, Aydın Adnan Menderes University, 09100 Aydın, Türkiye; 6 Instituto de Seguridad y Servicios Sociales de los Trabajadores del Estado (ISSSTE) Hospital Regional de Torreon, Hospital Regional de Torreón, Torreón 27000, Mexico; 7 Department of Pediatric Dentistry and Preclinical Dentistry, Wrocław Medical University, Krakowska 26 Street, 50-425 Wrocław, Poland; 8 Division of Histology and Embryology, Department of Biostructure and Animal Physiology, Wrocław University of Environmental and Life Sciences, Cypriana K. Norwida 25, 50-375 Wrocław, Polandpiotr.kuropka@upwr.edu.pl (P.K.)

**Keywords:** odontogenic myxoma, follicular cyst, juvenile cysts, odontogenic tumors, mandible myxoma

## Abstract

The occurrence of cysts and tumors in pediatric patients varies across different age groups. Follicular and dentigerous cysts are among the most common lesions. However, typical odontogenic tumors in juvenile patients are not frequently observed. Early stages of cyst and odontogenic tumor development might exhibit some similar characteristics due to the presence of unerupted teeth or their relationship with various stages of tooth formation and eruption. Many small lesions are discovered accidentally on routine orthopantomography (OPG), while the bigger ones manifest themselves as bone swelling, cortical perforation, or displacement and mobility of teeth. Each odontogenic tumor has characteristic clinical and radiological features. Biopsy of larger lesions, or incisional biopsy of smaller lesions, allows detailed histopathological evaluation to determine tumor type and growth behavior and guide appropriate treatment planning. In some cases, atypical signs on OPGs, like asymmetry in dental follicles, occurrence of round or oval bone lesions near impacted or retained teeth, and visibility of irregular radiolucent, radiopaque, or mixed jawbone lesions, might suggest the occurrence of some possible odontogenic tumor in juvenile patients. Each case should be handled individually. In this case, we demonstrate how atypical appearances of dental follicles on panoramic radiographs may not correspond with cone-beam computed tomography findings and may indicate the early stages of odontogenic myxoma in a juvenile patient.

## 1. Introduction

Odontogenic cysts and tumors can be found in the jawbones of different age groups [1,2]. While some are more common with typical radiological appearances, others might manifest themselves in different cases and might mimic other jaw lesions. The odontogenic myxoma (OM) is a quite rare benign odontogenic lesion. Its first description was introduced by Thoma and Goldman in 1974 [1,2]. Over the years, the WHO’s classification of odontogenic cysts and tumors changed frequently, while the description of the OM of a benign locally invasive and proliferative neoplasm in a mucoid background remained the same [1,2,3]. The estimation of OM occurrence ranges between 1 and 20% of each odontogenic tumor, while some sources report a 2–12% ratio [1,2,3,4,5,6,7]. OM origins are not yet fully confirmed; however, this lesion is more commonly found in young adults (second to third decade) with a slight female predominance in the tooth-bearing structures of the mandible. Clinically, OM is quite often asymptomatic and found accidentally; it grows slowly over time, is expansile, and may cause bone expansion and cortical destruction; it is quite often associated with tooth buds or impacted/unerupted teeth, mostly presenting a slow progressive bone lesion with only slight discomfort often experienced. OM, when examined, has mostly a gelatinous and loose structure, which quite often resembles myxomatous tumors, and then, a suspicion of jawbone myxoma could be expected [5,6,7,8,9,10]. A larger OM might cause pathological fractures, teeth resorption, jawbone and facial asymmetry and disfigurement, extracortical spread, and other symptoms associated with more advanced lesions. In each radiological study, its classic appearance is associated with multilocular radiolucency and with trabeculae arranged in a pattern known as a “tennis-racquet” or “step-ladder”, while some authors also suggest that, because of the scope of the radiological appearance, the “sun-ray” or “sun-burst” name can be used [1,2,3,4,5,6,7,8,9,10,11]. In small lesions, radiolucent areas may be easily misinterpreted as other cystic lesions or as an enlarged or asymmetric dental follicle.

**Figure 1:**
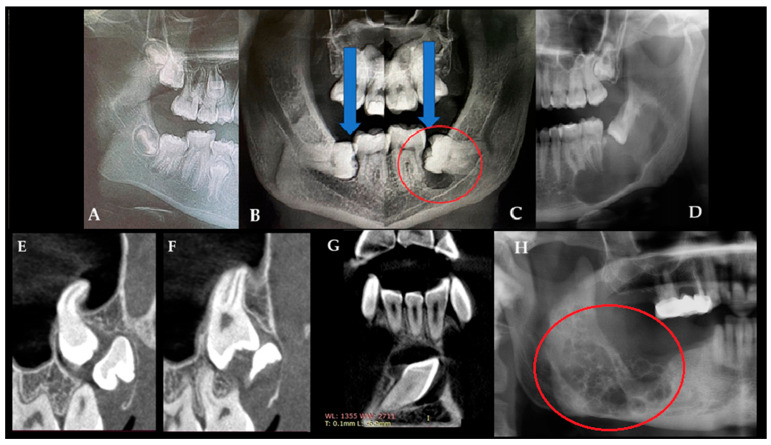
Many radiolucent lesions with well-defined borders represent cysts within the oral cavity. Each developing tooth is surrounded by a small, round radiolucent area known as the dental follicle, which is typically well defined and less than 4 mm in diameter (**A**). During growth, maturation, and tooth eruption, the dental follicle can remain, reduce its shape, disappear in time (blue arrows indicate the dental follicle on the left side that progressively regressed and disappeared), or form a follicular cyst (red circle). This situation occurs mostly when it is associated with totally or partially unerupted teeth (**B**,**C**). Panoramic radiography (OPG) is a useful initial screening tool and can identify various cystic and cyst-like jaw lesions of different sizes, shapes, and locations ((**D**)—unicystic ameloblastoma, UA). In general, retained teeth and associated cystic cavities should be further evaluated using CBCT (**E**–**G**). For comparison, a representative OPG of an odontogenic myxoma is shown in (**H**), demonstrating typical multilocular, lobulated (the red circle highlights the soap-bubble appearance) radiolucency with internal septations, a classic pattern that helps differentiate myxoma from simple cystic lesions. On the other hand, not all found cyst-like appearances on a routine OPG are a cyst; in these cases, CBCT is recommended to ensure that the dental follicle and formation of follicular cysts or any other cyst-like lesion do not have any worrisome symptoms, such as the following: bone erosion, teeth resorption, cortical swelling, any presence of bone irregularities, septa, or a mixed radiolucent–radiopaque appearance that might suggest the formation of a possible odontogenic tumor (abbreviation from image (**G**): I—inferior (lower border of mandible); WL/T (mm)—location of the follicle in CBCT in millimeters).

OPG is a useful initial screening tool for identifying bone cysts, lesions, and dental abnormalities. Quite often, a differentiation between dental follicles and typical cystic lesions of odontogenic and non-odontogenic origins can be assessed, as well as between solid lesions and other radiolucent, radiopaque, and mixed-appearance lesions [11,12]. Frequently, each cystic cavity in a juvenile patient should be differentiated between the following radiolucent cysts or cyst-like lesions: dentigerous cyst/follicular cyst (DC/FC), eruption cyst (EC), odontogenic keratocyst (OKC), unicystic ameloblastoma (UAM), radicular cyst, solitary bone cyst (SBC)/traumatic bone cyst, odontogenic myxoma (OM), or similar radiolucent unilocular lesions [9,10,11,12,13,14]. Routinely used OPG is quite a valuable diagnostic tool for finding any early-stage bone lesions, cysts, and tumors, and it can also be used for diagnostics with respect to teeth position, jawbone anomalies, and estimations of the dento-alveolar status of juvenile patients just before planned orthodontic treatment [15]. In cases of atypical bone architecture, such as a soap-bubble appearance, septated cystic lesions, calcifications, or irregular borders, cone-beam computed tomography (CBCT) is recommended to refine diagnoses and guide further diagnostic or surgical management. On the other hand, in juvenile patients, any enlargement or asymmetry in dental follicles surrounding retained or unerupted teeth might suggest the presence of a follicular cyst or indicate other tooth-related lesions. Sometimes, they can also cause odontogenic sinusitis when inflamed. It is worth identifying any asymmetry greater than 4 mm in any of the dental follicles and, if necessary, improving diagnostics with CBCT (Figure 1).

According to Bhardwaj et al., a dentigerous cyst (DC) is typically associated with the crown of an unerupted or partially erupted tooth and presents as a well-defined, asymptomatic, unilocular radiolucent lesion, which is sometimes accompanied by cortical expansion. Differential diagnosis should include radicular cyst, odontogenic keratocyst (OKC), ameloblastoma, ameloblastic fibroma, and odontoma [13,14]. CBCT provides a more detailed evaluation of jawbone lesions. In odontogenic tumors, particularly odontogenic myxoma (OM), characteristic CBCT features have been described, including internal septations forming triangular or rectangular spaces that create a “tennis-racket” or “honeycomb” appearance, scattered septa with or without tooth displacement or root resorption, and extension between dental roots with possible cortical breach and soft-tissue involvement [11,12,13,14]. During routine OPG screening, orthodontists can obtain valuable information not only about tooth position and jawbone relationships but also about the presence of potentially concerning bone lesions [15]. The role of CBCT is quite essential in evaluating the scope of bone changes, asymmetry, cortical perforation, resorption of dental apices, and the scope of bone invasion, as well as investigating the surrounding vital structures in detail, like the inferior alveolar nerve, maxillary sinus, and the proximity of dental buds [10,11,12,13,14,15].

This case highlights the role of OPG in early screening for the detection of odontogenic cysts and tumors, demonstrating how early identification can influence timely surgical intervention and improve patient outcomes.

## 2. Case Description

**Figure 2:**
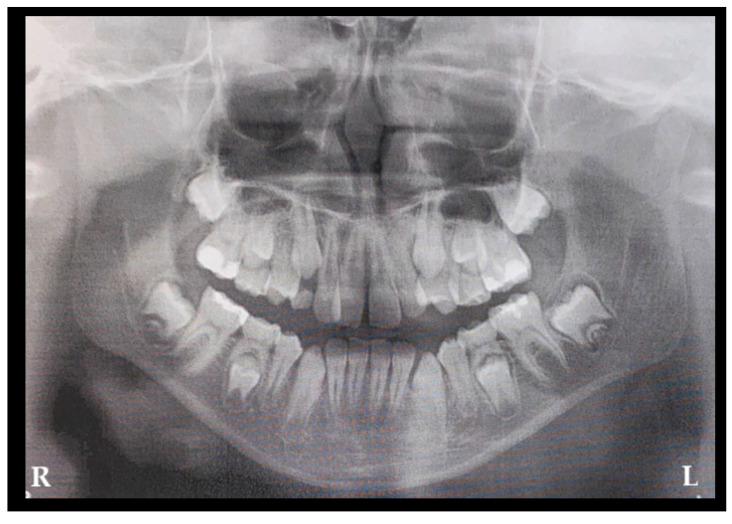
Initial scan—orthopantomogram (OPG) of a 13-year-old boy, consulted by a local orthodontist (abbreviations: R—right; L—left).

A generally healthy 13-year-old boy was referred for a routine orthodontic consultation. Medical history revealed no relevant conditions, including prior trauma, infection, or surgical treatment. At the start of orthodontic treatment, routine OPG screening demonstrated normal bone structure, mixed dentition, and appropriate jaw symmetry and alignment. Initial treatment included removable braces and oral hygiene (Figure 2: R—right side; L—left side). Routine OPG is a commonly used screening radiograph. In this case, it was performed as part of an orthodontic assessment to evaluate the presence and position of permanent teeth, the stage of dentition, and the presence of remaining deciduous teeth prior to further treatment. The bone structure of the jawbones is irrelevant in Figure 2. Initial orthodontic screening revealed no radiological signs of cysts and tumors. The shape and size of dental follicles surrounding both mandibular second permanent molars were normal. Bone structure was sustained, teeth buds were well positioned and symmetrical, and no worrisome radiological signs were present. The patient had no significant bone swelling, cortical expansion, or any significant changes in the intraoral examination.

**Figure 3:**
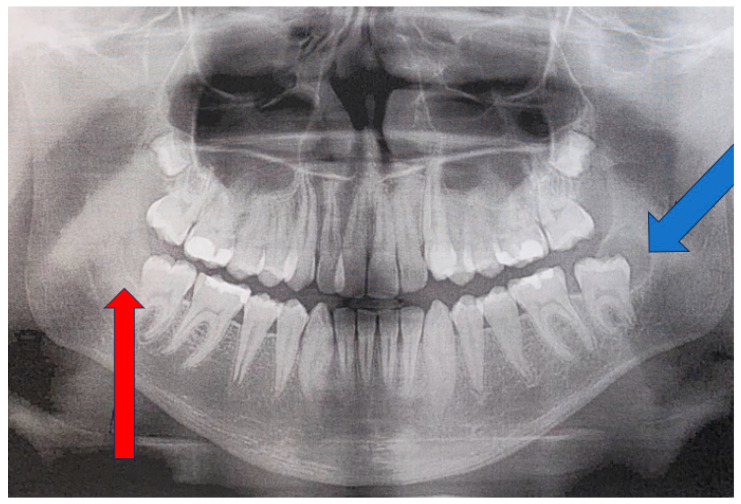
An OPG 18 months later with a visible, clearly defined radiolucent unilocular area behind the second left molar (blue arrow).

Control OPG radiograph after 18 months revealed the formation of a cystic lesion of radiolucent and unilocular appearance in the left retromolar area of the mandible (blue arrow). On the other hand, the right retromolar area was free of any disease, with normal bone contour and shape (red arrow). Because a left retromolar lesion was associated with a freshly, not fully yet erupted left mandibular second molar, an assumption of a follicular cyst (FC) was made. Since the lesion was unilocular and radiolucent, without any calcified or mineralized structures inside, a comparison with a cyst was made. In this case, the OPG showed no septations, ridges, or calcifications within the unilocular lesion (Figure 3). As the lesion progressed over time in the absence of clear clinical symptoms, CBCT imaging was performed to improve diagnostic assessment. If any suspicious findings, such as bone erosion, extracortical spread, resorption of adjacent teeth/roots, cortical bone erosion or swelling with a periosteal reaction, loss of bone structure with irregular borders, or other worrisome symptoms, are found on CBCT, a decision to perform either a biopsy or excisional biopsy should be made. The choice of approach depends on the lesion’s shape, size, and anatomical location within the jaw. The patient exhibited no worrisome swelling intraorally; their teeth were stable without any mobility, and bone asymmetry was not noted. In the following case, a needle biopsy did not reveal any fluid accumulation typical of a classic cyst of odontogenic origins.

**Figure 4:**
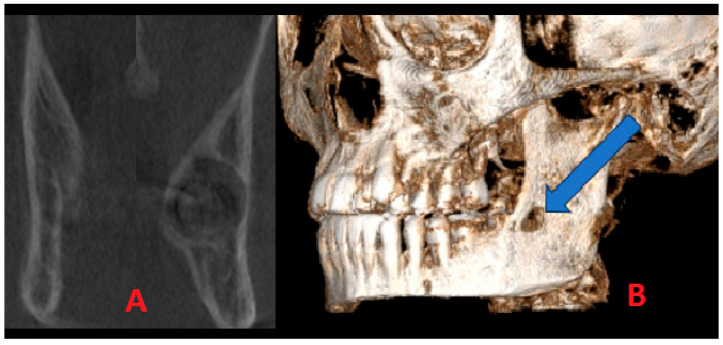
The CBCT visualization of the lesion: (**A**) bone asymmetry; (**B**) extracortical spread marked with blue arrow.

Additional CBCT was carried out to estimate the lesion’s boundaries and shape, the status of the cortical bone, and the proximity of the adjacent second molar, and the condition of the surrounding bones was visualized. A detailed CBCT evaluation of this cystic lesion revealed some worrisome radiological factors; however, no characteristic signs of OM were noted. This finding might be related to early OM detection in the early stages of growth and formation, and it is perhaps related to the remnants of the third molar germ or a bundle of not-yet-visible wisdom molar teeth. On the other hand, OM has a less unilocular appearance typically, which is more common for typical cystic lesions in this area. Coronal scans of both mandibular rami (A) revealed notable cortical enlargement and resorption to the lingual and buccal aspect on both sides of the cortical bone, with lesions penetrating towards the left mandible ramus. In this 3D reconstruction projection (B) of the lesion, clearly visible buccal cortical bone erosion and extra bone spread of the lesion were identified (Figure 4). Since the perforation was present, no typical “sunburst” appearance, characteristic of cortex perforation, and the presence of radiopaque lines extended from the periosteum was found. Another worrisome symptom in CBCT revealed significant bone swelling and asymmetry without the presence of the third molar. The lesion itself was very early in its initial stages of development; therefore, to assess its type and structure, a decision for early surgery was performed.

Given the radiological appearance and small size of the lesion, an excisional biopsy was scheduled. It should be noted that larger lesions showing radiographic signs of atypical behavior, such as root resorption, internal septations, honeycomb or sunburst patterns, or other concerning features, would initially require an incisional biopsy to establish the diagnosis before planning the definitive surgical approach.

Under general anesthesia, the lesion with a gel-like appearance, without any cyst lining, was removed. Additional soft tissues around the lesion were cut off as margins, and the entire bone cavity, as well as the distal aspect of the second molar teeth, was curetted. An additional burr-ostectomy was then planned to radicalize the procedure within all bony walls of the lesion, aiming to reduce the necessity of a secondary surgery and to improve surgical margins in the bone because of the gelatinous, spongy substance in the cystic lesion. No bone substitute material was used in the bone cavity; this was carried out only to compare and radiologically evaluate the scope of bone cavity healing after this radical procedure. Because of the gel-like nature of the lesion intraoperatively—lacking any solid or bone masses inside, with ill-defined borders and no typical cystic cavity lining—a suspicion of odontogenic myxoma (OM) was raised. In this case, due to the small size of the lesion, an excisional biopsy combined with curettage and burr osteotomy resulted in a favorable clinical outcome. The surgery and healing period were uneventful. In order to avoid any misdiagnosis with other odontogenic cysts and lesions, a careful histopathological evaluation was undertaken.

**Figure 5:**
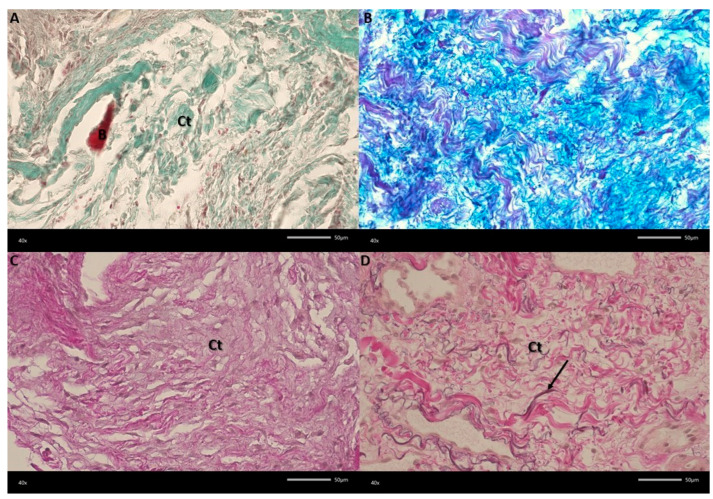
The histopathological specimen is presented in this figure (**A**–**D**).

The material was fixed for 72 h in a 4% buffered formaldehyde solution (pH 7.2–7.4). Tissues were washed in running water for 24 h, after which the sections were dehydrated by placing them in ethanol dilutions of increasing concentration. After impregnating them with paraffin, they were embedded in blocks. Tissues were cut into 7 μm thick sections and stained classically with hematoxylin and eosin (H&E), Alcian blue-PAS, Van Gieson for elastic fibers, and Masson–Goldner stain. All reagents were purchased from Merck KGaA (Darmstadt, Germany). The evaluation of histological preparations was performed using a Nikon Eclipse 80i microscope (Nikon, Tokyo, Japan) and a Jenoptik Gryphax^®^ Kapella camera with Gryphax^®^ software (JENOPTIK Optical Systems GmbH, Jena, Germany). With respect to the results, the center of the biopsy was filled with an abundant, loose, gelatinous extracellular matrix rich in mucopolysaccharides, giving it a strong blue appearance in Alcian blue staining (Figure 5). Between the sparse, thin collagen fibers, spindle-shaped, stellate, or round cells scattered throughout the stroma may be observed. In peripheral lesions, weak encapsulation is composed of thicker collagen fibers. In certain places, bone fragments from the alveolar process were observed, and elastic fibers were detected via Van Gieson staining. Compared to other tumors, a sparse vascular network was noted; however, the capillaries were surrounded by macrophages. No giant cells were visible in the changed tissues; therefore, giant cell granuloma was excluded. Figure 5A. A visible loose lesion structure consisting of green collagen fibers in tissue (Ct) and a small fragment of bone (B) was observed using the Mallory stain. Figure 5B. The presence of proteoglycans (blue) was observed during Alcian blue staining. Figure 5C. No elastic fibers in the lesion were observed using Van Gieson staining. Figure 5D. The collagen fibers are more densely packed in the adjacent area where elastic fibers are present (arrow) (Van Gieson stain (Mag 400×)). Moreover, from odontogenic fibroma, these findings differ due to the presence of sparse small collagen fibers, with the absence of elastic fibers immersed in an abundant gelatinous matrix, which is typical for immature tissue. No epithelial cells derived from odontogenic cells or glial cells can be noted; therefore, tumors such as ameloblastoma or myxoid neurofibroma are excluded.

**Figure 6:**
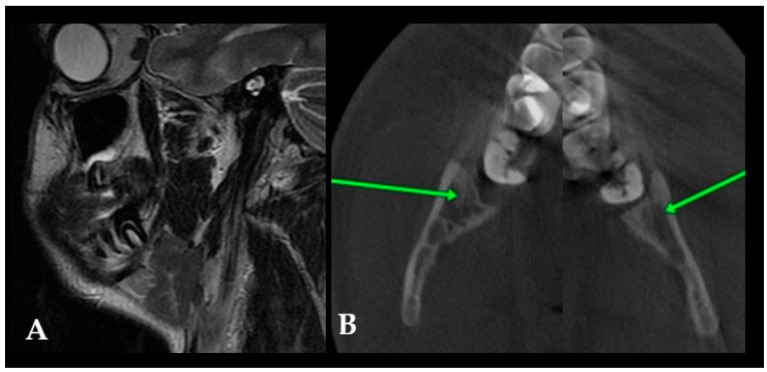
Postoperative magnetic resonance ((**A**), sagittal view) and CBCT (**B**) were conducted to assess bone healing. Green arrows ((**B**), axial view) point to good bone healing and proper bone structure after the procedure.

Postoperative MR (magnetic resonance) (A) with CBCT (B) after 12 months was used to evaluate the bone and adjacent soft tissue structure (Figure 6A). A satisfactory surgical outcome was achieved, with no radiographic evidence of recurrence or additional bone involvement. After surgery, a combination of MR-CBCT and MR-CT evaluations every six months for a year was conducted, and then, they were conducted once a year. This step was quite important for early detection of any relapse or secondary manifestation of OMs. The green arrows in Figure 6B indicate satisfactory bone healing, with comparable bone structures on both sides of the retromolar mandible. This surgical approach, consisting of biopsy combined with burr osteotomy, achieved a favorable outcome. This case underlines how OPG evaluation could be related to the early stages of this odontogenic tumor occurrence and its resemblance to an FC, growth patterns, and early detection during orthodontic screening. The intraoral approach did not reveal any worrisome findings in the patient.

**Figure 7:**
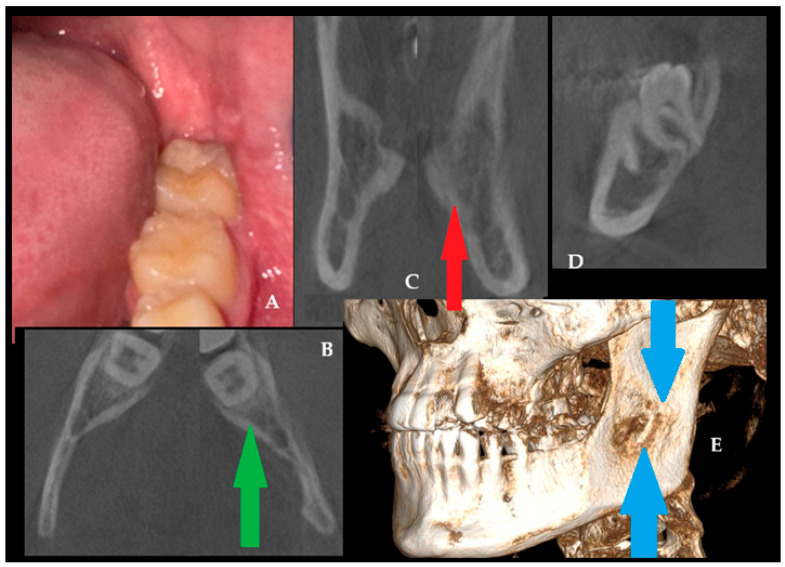
Healing results after a 24-month time frame (**A**,**B**). Bone swelling has disappeared ((**C**), red arrow), while the bone structure behind the second mandibular left molar is well restored (**B**,**D**), green arrow). Three-dimensional CBCT evaluation (blue arrows) demonstrates satisfactory bone healing and restoration of normal bone structure (**E**).

In this case (Figure 7), a favorable clinical (A) and radiological outcome was observed during the 24-month follow-up period. In a control CBCT axial view (green arrow, B), the bone healed without any asymmetry or swelling (red arrow, B). On the other hand, the previously reported asymmetry, swelling, and extracortical spread (Figure 4A) are not present, and the bone is totally healed with proper bone structure (Figure 7C, coronal view). The sagittal CBCT view (D) with CBCT-3D bone reconstruction (blue arrows, E) reveals the presence of cortical bone loss after a radical ostectomy of surgical bone margins, with clearly visible healing observed without any worrisome symptoms. This locally aggressive lesion requires good surgical planning and, later, a careful clinical and radiological evaluation of each patient during follow-up. The scope of each surgery should be focused on each case individually. CBCT helps identify and monitor patients closely. This case highlights how excisional biopsy, together with OPG findings refined by CBCT, facilitated accurate evaluations of the odontogenic myxoma [11]. The patient is still under observation, and routine follow-ups are scheduled every six months.

## 3. Discussion

The differentiation between a normal dental follicle, an enlarged and asymmetrical one, and inflamed follicles with possible cyst formation is quite important. Growing and undetected FC might affect bone shape, displace teeth, cause asymmetry, gingival swelling, and fistulas, and even lead to odontogenic sinusitis in some cases [8,9,10,11,12]. Therefore, a CBCT evaluation might improve patient outcomes and the prediction of the best surgical approach in each case.

The OM is a rare odontogenic tumor in juvenile patients. Most commonly, they are intraosseous benign lesions with an infiltrative character, and their sponge-like, gelatinous macroscopic structure is mostly related to abundant loose mucoid/myxoid stroma with few collagen elements, as evaluated microscopically [9,10,11]. The scope of each surgery is related to the lesion’s size and shape, the possible occurrence of a pseudo-cystic capsule, and OM spread within the spongious bone with or without clearly defined borders. Different factors affect the scope of radical surgery and the high percentage of tumor recurrence (5–25%) [7,8,9,10]. Careful evaluation of each lesion’s size, shape, and boundaries greatly affects the possible treatment approach. Authors like Scarfe et al. concluded that careful evaluation of each lesion in CT could affect not only possible lesion identification but also the diagnostic process, since some lesions’ typical radiological appearance could impact the diagnostic algorithm and pattern recognition of each bone lesion and therefore impact surgical planning [12]. Since a clear and round radiolucent unilocular area in OPG adjacent to each tooth might mimic a potential FC or any other similar cyst, the presumption of a GC, EC, or FC in each juvenile patient is more accurate when the presence of any septa, calcification, fibro-osseous, or similar features is not found [1,11,12,13]. Similarly to Shivashankara et al.’s study, OM in juvenile patients is not common, which also confirms the uniqueness of the presented case [10]. Each time, a good histopathological evaluation affects the final diagnosis. Sometimes, a myxoma can have more fibrous or fibre-like stroma components, and then, the identification of fibromyxoma (OFM) or very rarely an atypical case of fibromyxosarcoma (FMS) can be observed [2]. Juengsomjit et al. reported Bcl-2 immunoexpression in odontogenic myxoma; as an anti-apoptotic marker, Bcl-2 may contribute to tumor cell survival and may be related to tumor growth and locally aggressive behavior [8].

CBCT grants more valuable radiological data than a classic, routinely performed panoramic radiograph. Some worrisome radiological factors related to bone lesion irregularities and the presence of bone septa with or without some ossification within the cystic lesion, followed by teeth resorption, cortical swelling, and extracortical spread, might help in initial diagnostics. Furthermore, when a lesion is growing towards and within a maxillary sinus, some other sinus-related symptoms might occur. These can be more easily identified using CBCT rather than a classic radiograph. In the presented case, the asymmetry and increased radiolucent area behind a partially unerupted tooth indicated a follicular cyst. After improved diagnostics, CBCT followed by a histopathological specimen examination influenced the final diagnosis, especially because early OM growth patterns without any other radiological or clinical symptoms might mimic follicular cysts or merely the presence of a swollen, asymmetrical dental follicle.

In the presented case, with respect to OPG and CBCT, the early stages of OM could be easily misdiagnosed with other odontogenic cysts and tumors [2,3,4,5,6,7,8,9,10]. On the other hand, asymmetry in dental follicles and their volume of more than four millimeters might indicate the presence of an FC. Based on some of the studies, a single unilocular lesions (16.7% or less) are quite rare in OM [6,7,8,9]. The occurrence of mandibular unicystic, unilocular OM in juvenile patients is uncommon. Both CT and MR are essential for differential diagnosis in children and for monitoring bone involvement, particularly the spread of the lesion within bone cavities. According to Razek [11], both MR and CT can accurately investigate any changes in the trabecular bone and the spread of OM further into the bone. This intraosseous, benign, locally infiltrative neoplasm of the jawbones is mostly uncapsulated and occurs rarely in juvenile patients. Because surgical approaches vary widely in extent, reported recurrence rates range from approximately 5 to 10% and may reach 40–60% in some series [5,6,7,8,9,10,11,12,13,14]. The reported treatment strategies for OM range from conservative approaches (enucleation/curettage with or without peripheral ostectomy) to more radical procedures (marginal or segmental resection). The choice is typically guided by lesion size and location, radiographic extent (including cortical perforation or soft-tissue extension), proximity to vital structures, and the reported recurrence risk with conservative treatment [3,4,5,6,7,8,12,13,14,15,16]. Many studies indicate that simple enucleation of OM is insufficient because of the lesion structure itself. More radical approaches consisted of bone curettage, bone ostectomy, marginectomy procedures in the mandible, or partial/total resection of the affected bone, which is mostly associated with the diameter and location of each lesion [16,17,18,19,20,21,22,23,24,25]. Therefore, a careful radiological and clinical evaluation of each lesion affects the scope of each surgical approach. It appears that case-specific factors often affect this approach. Trode et al.’s study emphasizes that despite OM having a benign character, because of its aggressive and locally invasive nature, a more radical approaches are suggested to avoid any lesion relapse, mostly because of its high recurrence rate [5,6]. In the one of the studies, resection was the most common approach (65.2%), while the conservative approach was found in 34.8%, compared to other studies on average. A total recurrence of 35% was noted, while the highest recurrence was noted for a single OM enucleation (57.1%) [6,7,8,9,10,25,26]. Osman et al. reported a high recurrence rate of odontogenic myxoma and observed that a substantial proportion of patients had a history of tooth extraction related to the lesion [9]. On the other hand, Pacheco-Ojeda et al.’s study on a maxillary lesion, expanding outside the bone towards the palate, orbit, and adjacent soft tissues, reported requiring a more radical resection protocol [7]. Also, the resection protocol mentioned by Tavakoli and Williamson suggests that recurrence was never noted [22]. Regardless of the treatment approach, maintaining stable, disease-free bone is essential for preserving adequate occlusion. Some authors tend to leave the bone defect for secondary healing in small lesions, while others use one of the known reconstructive methods with or without titanium plates and screws to maintain the mandible’s proper shape and alignment. While some authors used allogenic or autologous bone transfers, some advised microsurgical reconstruction after resection or the usage of local flaps like the facial artery musculomucosal (FAMM), Bichat fat pads, or other local means to reconstruct the bone and soft tissue defect [20,21,22,23]. Regardless of all reviewed cases, a personalized decision for each patient seems to be the most commonly used. The authors do not consider routine radical resection to be necessary in all cases.

According to Oliveira et al.’s study, OM is mostly a well-defined osteolytic lesion with notable trabeculation within the lesion (soap-bubble, honeycomb, and tennis-racket appearances) [19]. While in this case swelling and asymmetry were not really visible, OM is mostly characterized by these symptoms [19]. On the other hand, Wang et al. studied 18 cases of OM patients using CBCT and concluded that CBCT is the most effective diagnostic tool for demonstrating OM cases [20]. The extent of surgery is largely case-dependent; however, due to the significant radiological and clinical overlap among jawbone lesions, histopathological examination remains the key determinant for definitive diagnosis and treatment planning [9,10,11]. Some studies suggest that more radical approaches are necessary because OM has a 25% recurrence rate, regardless of the fact that it is less commonly found in juvenile patients. It is worth remembering that each OM lesion progresses slowly in time, is symptomless, and can be found accidentally upon routine OPG screening. Rarely, atypical facial asymmetry, swelling, inflammation-like lesions, pain, or bone/facial deformity might be found in more advanced stages of OM. Unilocular lesions might be associated with impacted teeth, their displacement, and rarely, teeth resorption, or they can mimic any odontogenic germ and follicular cyst [20,21,22,23,24,25]. A review based on some Authors study on 61 cases revealed that tooth resorption was noted in 15.9%, tooth displacement in 54.5%, and cortical bone perforation in 38.7% of cases [20,21,22,23,24,25,26]. It is important to recognize that jaw lesions can present with a wide range of bone changes, varying in extent and severity. Osman et al. concluded that OM can mostly cause some asymptomatic bone expansion, and worrisome aspects like nerve paresthesia are uncommon (2.7%) [9]. This highlights the importance of high-quality radiological imaging in the evaluation of jaw lesions [20,21,22,23,24,25].

The following lessons can be drawn from this case: enlarged dental follicles are not always cystic; radiological signs of bone erosion warrant further investigation; early cysts and tumors in children may appear similar; the absence of fluid on needle aspiration should prompt biopsy; early OM might radiographically mimic FC; CBCT is especially useful for evaluating cortical bone and tooth involvement; a combination of CBCT and histopathology are critical for correct diagnosis.

## 4. Conclusions

The surgical approach to each odontogenic myxoma case is very much case-related. A conservative approach to the lesions is related to a higher risk of local recurrence. In juvenile patients, the initial growth of OM might mimic a swollen dental follicle or the presence of a follicular cyst. Early detected and locally advanced lesions are treated with overall higher success rates and less invasive surgical approaches. The extent of surgical resection should be greater in cases with more advanced lesions, progressive bone loss, extracortical spread towards soft tissues, or those causing facial disfigurements. In the presented case, we emphasize the role of early OPG detection and the use of CBCT in surgical planning, which resulted in an overall good final result.

## Data Availability

The datasets used and/or analyzed during the current study are available from the corresponding author upon reasonable request.

## References

[1] Soluk-Tekkesin M., Wright J.M. (2022). The World Health Organization Classification of Odontogenic Lesions: A Summary of the Changes of the 2022 (5th) Edition. Turk. J. Pathol..

[2] Banasser A.M., Bawazir M.M., Islam M.N., Bhattacharyya I., Cohen D.M., Fitzpatrick S.G. (2020). Odontogenic Myxoma: A 23-Year Retrospective Series of 38 Cases. Head Neck Pathol..

[3] Gupta S., Grover N., Kadam A., Gupta S., Sah K., Sunitha J.D. (2013). Odontogenic myxoma. Natl. J. Maxillofac. Surg..

[4] Vladić M.B., Vuletić M., Seiwerth S., Gabrić D. (2025). Odontogenic Myxoma in the Anterior Part of the Mandible—A Case Report. Surgeries.

[5] Trode H., Pouget C., Talbi M., Simon E., Brix M. (2023). Surgical management of odontogenic myxomas: A case series. Int. J. Surg. Case Rep..

[6] Nguyen T.T.H., Eo M.Y., Cho Y.J., Myoung H., Kim S.M. (2021). Large myxomatous odontogenic tumor in the jaw: A case series. J. Korean Assoc. Oral Maxillofac. Surg..

[7] Pacheco-Ojeda L., Díaz-Yépez M., Castillo-Aguirre G., Mogrovejo-Freire L., Cañizares-Quisiguiña S. (2025). Aggressive vascularized odontogenic myxoma. Case report and literature review. Int. J. Surg. Case Rep..

[8] Juengsomjit R., Arayasantiparb R., Ghazali A.B., Kosanwat T. (2024). Odontogenic myxoma: A clinicopathological study over 15 years and immunohistochemical analysis. Heliyon.

[9] Osman S., Hamouda G.M., Eltohami Y.I. (2024). Clinical Spectrum and Treatment of Odontogenic Myxoma: Analysis of 37 Cases. J. Maxillofac. Oral Surg..

[10] Shivashankara C., Nidoni M., Patil S., Shashikala K.T. (2017). Odontogenic myxoma: A review with report of an uncommon case with recurrence in the mandible of a teenage male. Saudi Dent. J..

[11] Abdel Razek A.A.K. (2019). Odontogenic Tumors: Imaging-Based Review of the Fourth Edition of World Health Organization Classification. J. Comput. Assist. Tomogr..

[12] Scarfe W.C., Toghyani S., Azevedo B. (2018). Imaging of Benign Odontogenic Lesions. Radiol. Clin. N. Am..

[13] Bhardwaj B., Sharma S., Chitlangia P., Agarwal P., Bhamboo A., Rastogi K. (2016). Mandibular Dentigerous Cyst in a 10-Year-Old Child. Int. J. Clin. Pediatr. Dent..

[14] Devenney-Cakir B., Subramaniam R.M., Reddy S.M., Imsande H., Gohel A., Sakai O. (2011). Cystic and cystic-appearing lesions of the mandible: Review. Am. J. Roentgenol..

[15] Smołka P., Nelke K., Struzik N., Wiśniewska K., Kiryk S., Kensy J., Dobrzyński W., Kiryk J., Matys J., Dobrzyński M. (2024). Discrepancies in Cephalometric Analysis Results between Orthodontists and Radiologists and Artificial Intelligence: A Systematic Review. Appl. Sci..

[16] Dalbo Contrera Toro M., Siqueira Barreto I., Amstalden E.M., Takahiro Chone C., Nizam Pfeilsticker L. (2016). Odontogenic Myxoma in Children: A Case Report and Literature Review. Case Rep. Oncol. Med..

[17] Lis E., Gontarz M., Marecik T., Wyszyńska-Pawelec G., Bargiel J. (2024). Residual Cyst Mimicking an Aggressive Neoplasm—A Life-Threatening Condition. Oral.

[18] Ghazali A.B., Arayasantiparb R., Juengsomjit R., Lam-Ubol A. (2021). Central Odontogenic Myxoma: A Radiographic Analysis. Int. J. Dent..

[19] Oliveira S.V., Rocha A.C., Ceccheti M.M., Gallo C.B., Alves F.A. (2018). Odontogenic myxoma in a child treated with enucleation and curettage. Autops. Case Rep..

[20] Wang K., Guo W., You M., Liu L., Tang B., Zheng G. (2017). Characteristic features of the odontogenic myxoma on cone beam computed tomography. Dentomaxillofacial Radiol..

[21] Gontarz M., Bargiel J., Gąsiorowski K., Marecik T., Szczurowski P., Zapała J., Wyszyńska-Pawelec G. (2021). Extended, Double-Pedicled Facial Artery Musculomucosal (dpFAMM) Flap in Tongue Reconstruction in Edentulous Patients: Preliminary Report and Flap Design. Medicina.

[22] Tavakoli M., Williamson R. (2019). Odontogenic myxomas: What is the ideal treatment?. BMJ Case Rep..

[23] Khalil A., Ahmad K.G., Khalil M., Salloum R. (2023). Odontogenic myxoma of the mandible: A case report. Ann. Med. Surg..

[24] Duś-Szachniewicz K., Woźniak M., Nelke K., Gamian E., Gerber H., Ziółkowski P. (2015). Protein tyrosine phosphatase receptor R and Z1 expression as independent prognostic indicators in oral squamous cell carcinoma. Head Neck.

[25] McLean A.C., Vargas P.A. (2023). Cystic Lesions of the Jaws: The Top 10 Differential Diagnoses to Ponder. Head Neck Pathol..

[26] Boffano P., Stathopoulos P., Ruslin M. (2025). Pathological and Molecular Features of Odontogenic Myxoma: A Systematic Review. Indian J. Otolaryngol. Head Neck Surg..

